# A rare case of type 1 aortic dissection with carotid arter occlusion, preoperative seizure, and postoperative spinal cord ischemia

**DOI:** 10.1186/s43044-026-00732-9

**Published:** 2026-03-30

**Authors:** Mehmet Işık, Hanefi Furkan Öz, Hasan Hüseyin Kır, Yalçın Günerhan, Yüksel Dereli

**Affiliations:** Necmettin Erbakan University, Konya, Turkey

**Keywords:** Type 1 aortic dissection, Cardiovascular surgery, Neurological findings

## Abstract

**Background:**

Aortic dissections are cardiovascular cases with high mortality that can present with many different clinical findings. The development of preoperative neurological findings may negatively affect postoperative results.

**Case presentation:**

In this case, a patient with a right common carotid artery occlusion due to type 1 aortic dissection, a plegic right lowers extremity, and a generalized tonic-clonic seizure just before surgery was presented. After the Bentall operation, spinal cord ischemia was diagnosed and pulse steroid treatment was started.

**Conclusions:**

The case was wanted to be shared due to its specific neurological and vascular condition.

## Introduction

Aortic dissection (AD) occurs as a result of a tear between the intima and media of the aorta. The main symptom is severe back and/or chest pain. Depending on the extent of the dissection, abdominal pain, neurological findings, extremity ischemia and blood pressure differences may be observed [1, 2]. The most common etiological cause is hypertension [2]. The annual incidence of aortic dissection has been reported as 4.6 per 100,000 [3]. Although it can occur in all age groups, it is seen in 75% of males between the ages of 40–70 [1]. While the average mortality rate during surgical intervention is 22%, the total in-hospital mortality rate is approximately 30% [4].

In this case, a patient with a right common carotid artery occlusion due to type 1 aortic dissection, a plegic right lowers extremity, and a generalized tonic-clonic seizure just before surgery was presented. After the Bentall operation, spinal cord ischemia was diagnosed and pulse steroid treatment was started. The case was wanted to be shared due to its specific neurological and vascular condition.

## Case

**Fig. 1:**
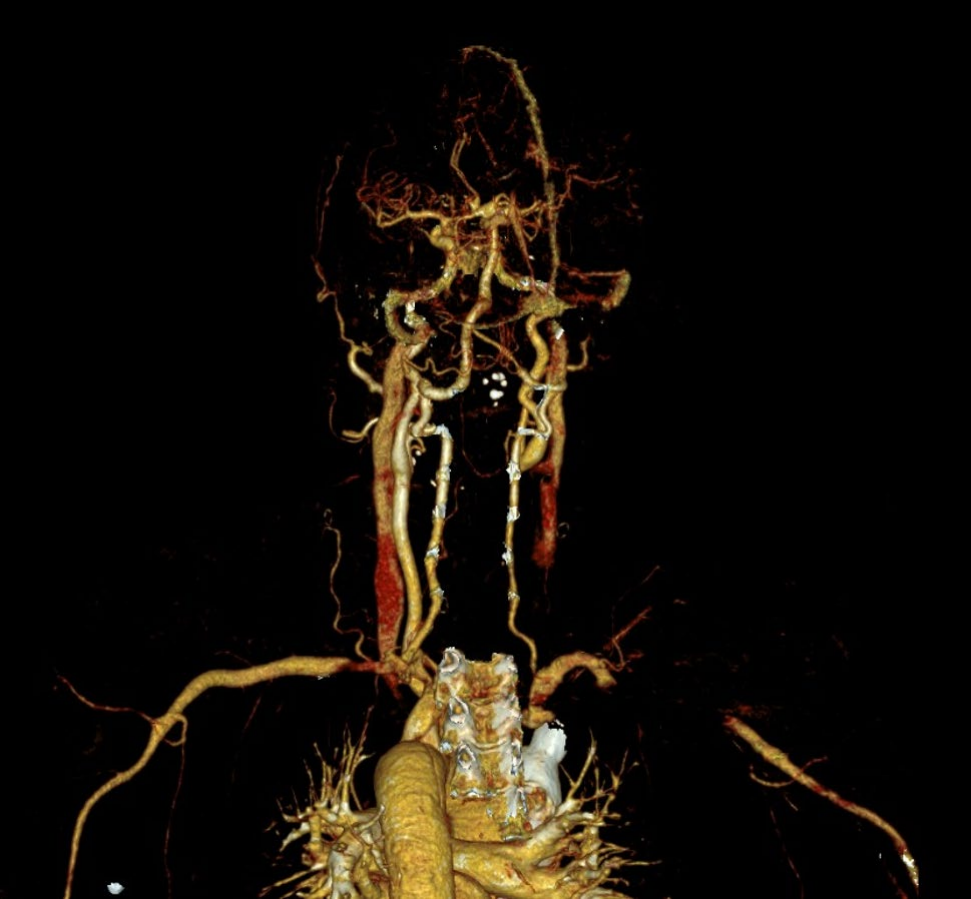
Thromboses right carotid artery

A 50-year-old male patient presented to the emergency department with right leg weakness, loss of sensation, and transient speech disorder that began half an hour ago. He had a history of hypertension and a 70-pack-year smoking history. On physical examination, he was conscious, oriented, cooperative, the blood pressure difference between the right and left arms was 45 mmHg, and the right lower extremity was plegic. Other physical examination findings were normal. Computed tomography (CT) angiography showed a type 1 aortic dissection extending to the iliac bifurcation. The dissection also extended to the right brachiocephalic artery and left subclavian artery. The right subclavian and left common carotid arteries originated from the true lumen and showed normal filling. The right common carotid artery originated from the false lumen and no flow was observed in its lumen (occlusion ?) (Fig. 1).

**Fig. 2:**
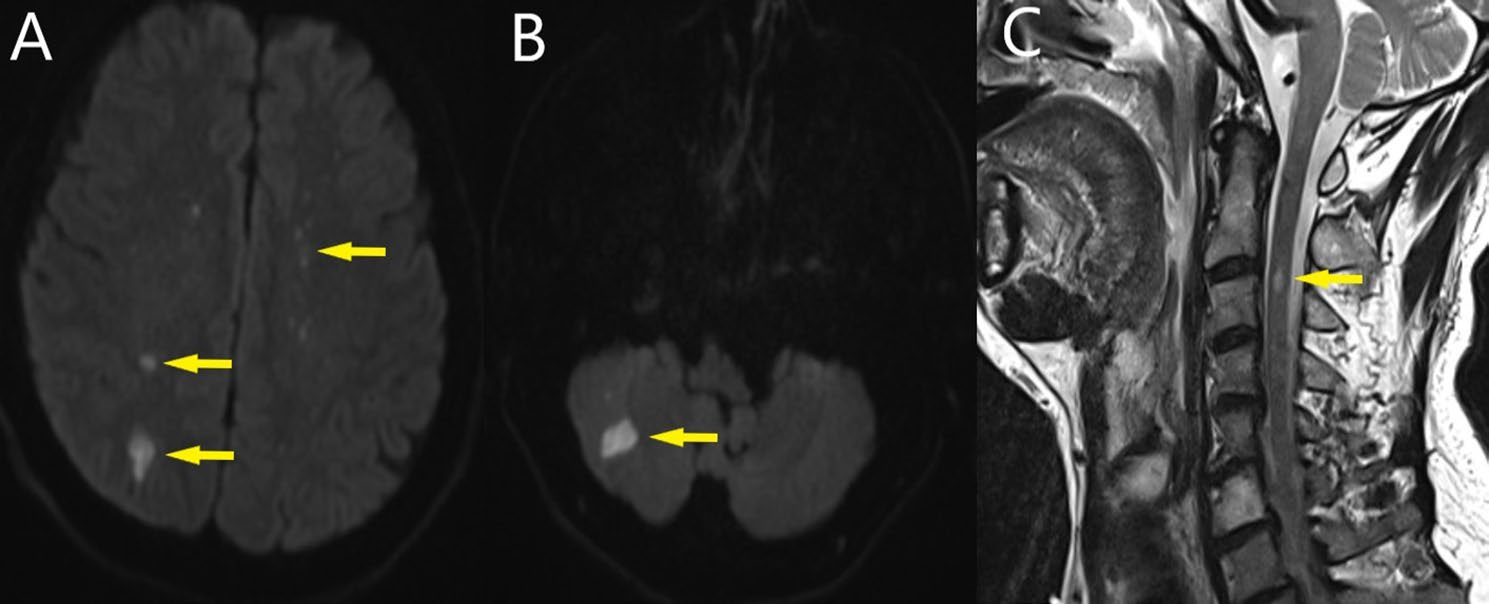
**A**, **B** Small diffusion restrictions, **C** hyperintense lesion in spinal cord

Echocardiography report was as follows; EF 60%, severe aortic insufficiency, ascending aorta 41 mm, no signs of compression and dissection flap image was observed in ascending aorta. While preparing for emergency operation, the patient had a generalized tonic-clonic seizure lasting approximately 1 min. During the surgery, Bentall procedure and hemiarcus replacement (with a 25 no. mechanical prostatic aortic valve conduit) were performed. Since a flap was seen in the inner curvature of the aortic arch, total circulatory arrest lasting 28 min was applied. Retrograde cerebral perfusion (via the superior vena cava, with a flow of 500 cc/min) was performed during total circulatory arrest. During retrograde perfusion, there was backflow from the right brachiocephalic artery. During intensive care follow-up, the patient’s sedation was discontinued at the 5th postoperative hour. In the neurological examination four hours after sedation was stopped; consciousness tended to be slightly sleepy, eye opening response was present with verbal stimulus, simple commands were followed, facial asymmetry was absent, light reflex was bilaterally normal and pupils were isochoric. Muscle strength was 1/5 with painful stimulus in bilateral upper extremities and 3/5 with painful stimulus in bilateral lower extremities. Babinski and Hoffman signs were evaluated as bilaterally negative. As a result of neurology consultation, cervical and brain magnetic resonance imaging (MRI) was performed to screen for vascular pathologies of the cerebral and cervical spinal cord. In diffusion MRI images, diffusion restriction consistent with millimetric widespread acute infarction was observed in both cerebral hemispheres, especially at the vertex level, ACA, MCA transition zone level, right occipitoparietal level, and bilateral cerebellar hemispheres (Fig. 2A, B). In cervical MRI images, a hyperintense lesion was observed in the spinal cord at the C2 vertebra level in sagittal T2 weighted images (Fig. 2C). As a result of radiological images, bilateral cerebral embolism and spinal cord ischemia at the C2 vertebra level were considered as probable diagnoses. In addition to antiaggregant and anticoagulation therapy, 250 mg IV prednisolone treatment was started at 06:00 for 5 days in accordance with the recommendation of neurology. The patient was extubated according to blood gas values ​​on the 2nd postoperative day (extubation was delayed due to intense secretions). His hemodynamics remained stable during follow-ups and he was transferred to the physical therapy and rehabilitation clinic on the 19th postoperative day due to loss of upper extremity strength.

## Discussion

While the most common symptoms of type 1 aortic dissection are chest and back pain, it can also present with isolated neurological symptoms or extremity findings [5, 6]. In this case, the presenting complaints included transient speech impairment and loss of muscle strength in the right lower extremity. Due to the patient’s complaints, neurological pre-diagnoses were considered during the first examination in the emergency department. Since type 1 aortic dissections can affect all segments of the aorta, they can present with different clinical findings. The most frequently confused differential diagnoses are myocardial infarction and stroke [2]. Different clinical findings make diagnosis difficult and prolong the time until surgery. In this case, the time between the onset of the patient’s complaints and the time he was taken to surgery was 5.5 h. Many studies have reported that early diagnosis and urgent surgical intervention in AD are among the most important factors that reduce mortality [2, 4].

In a patient who will undergo new surgery, the presence of acute neurological symptoms is a condition that is thought to negatively affect postoperative results. In this case, there was weakness in the right lower extremity before the operation. This is likely related to the dissection flap at the iliac bifurcation. Furthermore, the patient experienced a generalized tonic-clonic seizure during the operation. Preoperative CT angiography revealed no major findings to explain the neurological symptoms other than right carotid artery occlusion. In the postoperative period, the weakness in the right lower extremity decreased to 3/5 and no new findings that could be defined as seizures were observed. This improvement seen in the early period may be related to the remodeling of the aorta after the Bentall and hemiarcus operation.

However, postoperatively, tetraparesis, more prominent in bilateral upper extremities, occurred. As a result of neurological evaluation and imaging, a diagnosis of cerebral multiple millimetric infarcts and spinal cord ischemia at the C2 vertebra level was made. Spinal cord ischemia and millimetric cerebral infarctions may be associated with low perfusion pressure, total circulatory arrest, and microembolic events during surgery.

The detection of preoperative neurological symptoms in the patient led us to re-evaluate neurologically in the early postoperative period. Neurology consultation was also performed and early pulse steroid and antithrombotic treatment was started for spinal cord ischemia. Regression of neurological symptoms may be related to early diagnosis and treatment.

## Conclusion

This case illustrates the diagnostic complexity and potential for severe neurological complications in type I aortic dissection. The presence of neurological symptoms warrants urgent imaging and multidisciplinary evaluation. Both preoperative and postoperative neurological monitoring are essential to optimize outcomes in such high-risk patients.

## Data Availability

No datasets were generated or analysed during the current study.

## Abbreviations

AD: Aortic dissection
CT: Computed tomography
EF: Ejection fraction
MRI: Magnetic resonance imaging
ACA: Anterior cerebral artery
MCA: Middle cerebral artery
